# Carboxypeptidase A4 negatively correlates with p53 expression and regulates the stemness of breast cancer cells

**DOI:** 10.7150/ijms.54954

**Published:** 2021-02-18

**Authors:** Yipeng Wang, Yibin Xie, Yanan Niu, Peng Song, Ye Liu, Joseph Burnett, Zhihua Yang, Duxin Sun, Yuliang Ran, Yang Li, Lichao Sun

**Affiliations:** 1 Department of Breast Surgery, National Cancer Center/National Clinical Research Center for Cancer/Cancer Hospital, Chinese Academy of Medical Sciences and Peking Union Medical College, Beijing 100021, China.; 2 State Key Laboratory of Molecular Oncology, National Cancer Center/National Clinical Research Center for Cancer/Cancer Hospital, Chinese Academy of Medical Sciences and Peking Union Medical College, Beijing, 100021, P.R. China.; 3 Department of Pancreatic and Gastric Surgery, National Cancer Center/National Clinical Research Center for Cancer/Cancer Hospital, Chinese Academy of Medical Sciences and Peking Union Medical College, Beijing 100021, P.R. China.; 4 Department of oncology, second medical centre of Chinese PLA General Hospital, Beijing, P.R. China.; 5 Department of Pharmaceutical Sciences, University of Michigan, Ann Arbor, MI 48109.; 6 Department of Anesthesiology, Beijing Obstetrics and Gynecology Hospital, Capital Medical University, Beijing, P.R. China.; 7 Institute of Medical Information, Chinese Academy of Medical Sciences and Peking Union Medical College, Beijing 100020, P.R. China.

**Keywords:** triple negative breast cancer, CPA4, P53, biomarker, stemness

## Abstract

**Background:** Triple-negative breast cancer (TNBC) is an aggressive cancer subtype lacking effective treatment options, and p53 is the most frequently mutated or deleted gene. Carboxypeptidase A4 (CPA4) is an extracellular metallocarboxypeptidase, which was closely associated with aggressiveness. Although a recent study indicated that CPA4 could induce epithelial‑mesenchymal transition in breast cancer cells, no studies investigated its stemness-related function and the correlation between CPA4 and p53 in TNBC. In this study, we aimed to investigate the CPA4 levels in breast cancer tissues and analyze its association with p53, and study its roles in cancer stemness maintenance.

**Methods:** CPA4 mRNA level and its prognostic value were analyzed by using online database UALCAN (http://ualcan.path.uab.edu) and Kaplan-Meier plotter (www.kmplot.com), respectively. The expression of CPA4, p53 and ALDH1A1 in breast cancer and adjacent normal tissues were evaluated by IHC using the corresponding primary antibodies on a commercial tissue array (Shanghai Biochip Co., Ltd., Shanghai, China). siRNA knockdown was used to study the function of proliferation, colony formation assay and sphere formation in serum-free medium.

**Results:** Analysis of the UALCAN datasets identified that CPA4 mRNA levels were elevated in TNBC, especially in the TP53-mutant subgroup. Furthermore, high levels of CPA4 mRNA were significantly associated with unfavourable overall survival OS in breast cancer patients. Immunohistochemistical analysis demonstrated that CPA4 levels were elevated in 32.1% of breast cancer samples (45/140), and the positive rates of ALDH1A1 and p53 in the breast cancer tissues were 25% (35/140) and 50% (70/140), respectively. Statistical analysis revealed high levels of CPA4 was significantly associated with TNBC phenotype. Correlation analysis indicated that CPA4 over-expression was positively associated with ALDH1A1 (P<0.01) and negatively correlated with p53 (P<0.05). In Kaplan-Meier survival analysis, either high CPA4 or ALDH1A1 levels was significantly correlated with poor survival in breast cancer patients. Functional studies demonstrated that down-regulation of CPA4 significantly inhibited TNBC cell proliferation, colony-formation assays in soft agar and sphere formation in serum-free medium.

**Conclusion:** This study demonstrated for the first time that CPA4 was negatively correlates with p53 expression and inhibition of CPA4 could reduce the number of breast cancer cells with stemness property. It might be a potential target for the TNBC treatment.

## Introduction

Triple-negative breast cancer (TNBC) is an aggressive breast cancer subtype that accounts for 15%-20% of all breast cancers. The tumor suppressor, p53, is frequently lost in breast cancer patients. Although tremendous advances have been made in treating other breast cancer subtypes, TNBC still lacked effective treatment options, and always developed the drug resistance, cancer relapse, or distant metastasis.

The cancer stem cell (CSC) hypothesis suggests that many cancers are sustained by a small population of CSCs, which have the ability to self-renew, differentiate and generate daughter cancer cells. CSCs were thought to be responsible for cancer recurrence and metastasis. Cancer stem cell could be recognized by markers, and Aldehyde dehydrogenase 1A1 (ALDH1A1) is a candidate breast CSC marker 1. A great many of evidences indicated that CSC were enriched in TNBC. Our recent study showed that breast cancer cell line BT474 would undergo epithelial mesenchymal transition (EMT) and possess triple negative phenotype (BT474-PTEN-LTT, LTT) after trastuzumab treatment^2^. Then we employed RNA-seq to identify the potential genes involving this process, and Carboxypeptidase A4 (CPA4) was selected as the top candidate gene, and p53 was also found to be down-regulated in LTT cells^3^. CPA4 is a zinc-dependent peptidolytic enzyme. Our previous studies firstly demonstrated that CPA4 was over-expressed in pancreatic, liver, esophageal and lung cancer tissues^4^-^8^. Additionally, aberrant CPA4 expression was closely associated with tumor progression^9^, ^10^. However, few studies investigated its stemness-related function and the correlation between CPA4 and p53 in breast cancer. In this study, we aimed to investigate the association between CPA4 and p53 in breast cancer tissues, and study its roles in cancer stemness maintenance.

## Methods

### UALCAN database analysis

UALCAN database (http://ualcan.path.uab.edu) was used for analyzing cancer transcriptome data from The Cancer Genome Atlas (TCGA). The prognostic significance of the mRNA levels of CPA4 in breast cancer was evaluated by using the Kaplan-Meier plotter (www.kmplot.com).

### Immunohistochemistry and tissue microarray assay

The commercial tissue array containing 140 cases of breast cancer patients were constructed by Shanghai Biochip Co. Ltd. as described. For all the specimens, clinicopathological information (Age, Gender, Grade, TNM stage, and follow-up data) was available. The expression of CPA4, p53 and ALDH1A1 in the tissues were evaluated by immunohistochemical staining with specific antibodies. According to UltrasensitiveTM S-P immunohistochemistry kit protocol (Maixin Biotechnology Development Co., Ltd.), standard Avidin-biotin complex peroxidase immunohistochemical staining was performed. Briefly, after deparaffinizationin xylene and graded alcohols, heated antigen retrieval was done in citrate buffer (10mmol/L pH 6.0) by water-bath kettle heating for 30min. Endogenous peroxidase was blocked in 0.3% hydrogen peroxide for 10 min. Nonspecific binding was blocked by incubation in 10% normal animal serum for 10min. Sections were incubated at 4°C for 24 h with primary antibodies including polyclonal antibody against anti-CPA4 (HPA021030, Sigma-Aldrich), ALDH1A1 (Abcam, ab52492) and anti-p53 antibody (ab28, Abcam). Biotinylated secondary antibody and horseradish peroxidase-labeled avidin were subsequently used, and color was developed using the diaminobenzidine method.

### Immunostaining analysis

Slides were independently evaluated by 2 two pathologists who were blinded to patients' clinical data. Protein expression levels were scored by staining intensity and the percentage of immunoreactive cancer cells. Staining intensity was arbitrarily scored on a scale of four grades: 0 (no staining of cancer cells or weak staining), 1 (moderate staining), 2 (moderate staining), and 3(strong staining). For CPA4, the percentage of positive cells was scored as follows: 0 (0%), 1 (1% to 30%), 2 (31% to 50%), and 3 (>50%). For ALDH1A1, the percentage of positive cells was scored as follows: 0 (0%), 1 (1% to 5%), 2 (5% to 40%), and 3 (>40%). For p53, the percentage of positive cells was scored as follows: 0 (0%), 1 (1% to 10%), 2 (11% to 50%), and 3 (>50%). The staining positivity was determined using the following formula: overall score=positive percentage score x intensity score. For CPA4, a score of 0 to≤4 was defined as “0”, and >4 as “1”. For ALDH1A1, a score of 0 to≤2 was defined as “0”, and >2 as “1”. For p53, a score of 0 t o≤1 was defined as "0, Negative", and >1 as “1, Positive”.

### Knockdown by siRNA

Small interfering RNAs for gene CPA4 were purchased from Qiagen (validated FlexiTube siRNA SI00115605). Transfection of BT474 PTEN-LTT cells was carried out using Lipofectamine® RNAiMAX vehicle according to the manufacturer's instruction following optimization. As a negative control, a non-targeting sequence siRNA was utilized (Qiagen, catalog number 1027281). Knockdown at mRNA level was confirmed by isolating total RNA (RNeasy Mini kit, Qiagen) and performing real-time quantitative RT-PCR in triplicate. Real-time PCR was carried out on an ABI PRISM 7900HT sequence detection system (Applied Biosystems).

### MTS cell proliferation assay

Cancer cells were seeded at a density of 2,000 cells per well in 96-well plates and allowed to adhere overnight. Cell growth was determined by MTS assay according to manufacturer's instruction by measuring the absorbance at 490 nm on a Synergy 2 plate reader (Biotek).

### Mammosphere formation assay

Mammosphere culture was done according to MammoCult™ Human Medium Kit (05620, STEMCELL Technologies Inc.) supplemented with Heparin and Hydrocortisone. Single cells were prepared from mechanical and enzymatic dissociation were plated in six-well ultralow attachment plates (Corning) at a density of 500 cells/ml. After 14 days of culture, the number of mammospheres was counted on a Nikon Eclipse TE2000-S microscope and the photos were acquired with MetaMorph 7.6.0.0.

### Statistical analysis

The SPSS 15 software package (SPSS, Inc., Chicago, IL) was used for statistical analysis. The association between the markers and clinicopathologic features was analyzed using χ2-test or two-sided t-test as appropriate. Survival curves were calculated using the Kaplan‑Meier method and compared by the log-rank test. Spearman's rank correlation coefficient and Fisher's exact test were used to explore the association between CPA4 and ALDH1A1 expression. All the comparisons were conducted using two-tailed, and p<0.05 was considered statistically significant.

## Results

### Association of CPA4 mRNA expression with clinicopathological parameters of breast cancer patients

**Figure 1:**
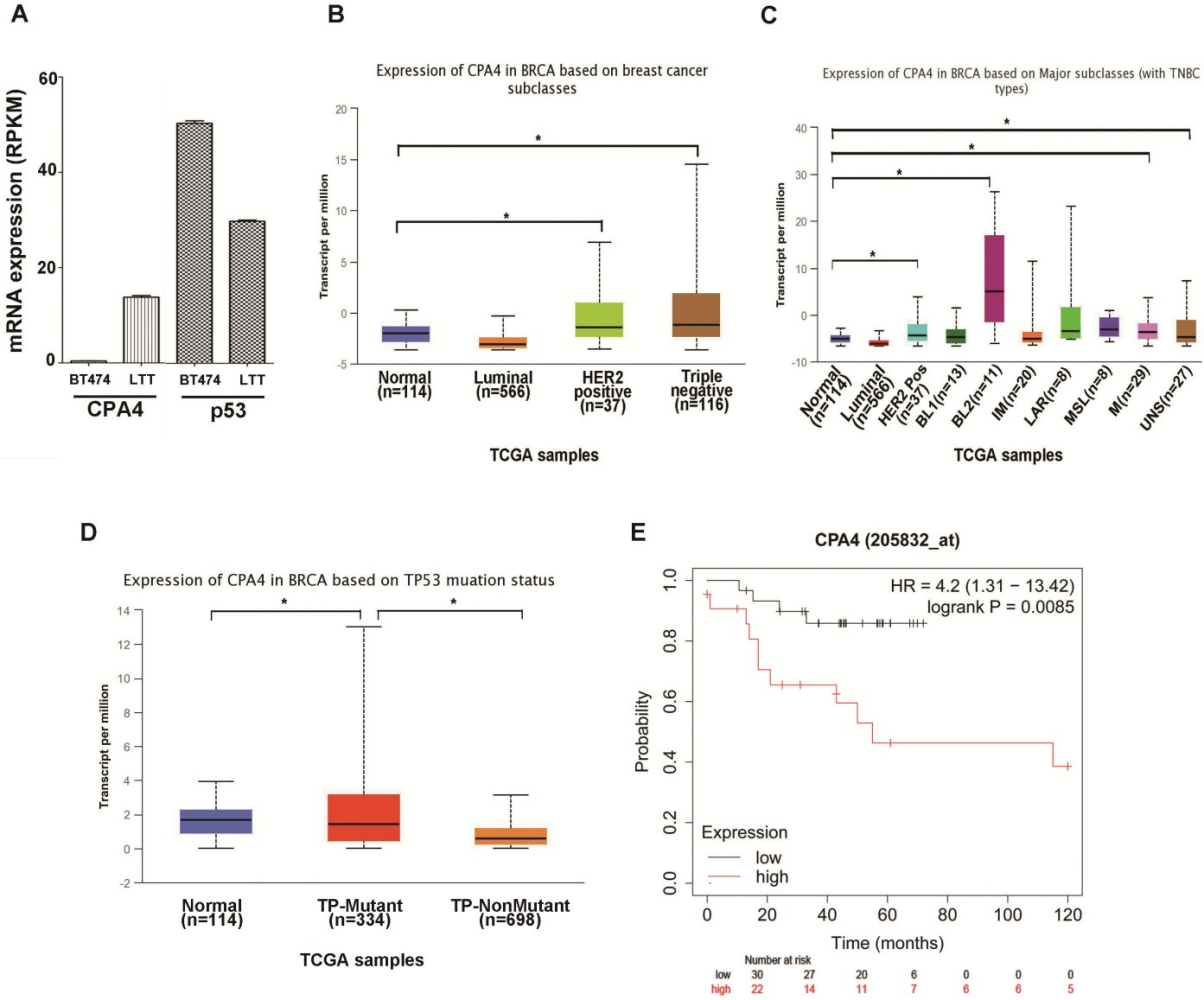
** UALCAN analysis of the correlation between CPA4 mRNA expression levels and clinicopathological parameters of breast cancer. (A)** mRNA expression of CPA4 and p53 in BT474 and LTT cells. **(B)** Breast cancer subclass (luminal, HER2+, and triple-negative). **(C)** Breast cancer subclass with TNBC subclass. **(D)** Breast cancer with p53 mutant or p53 non-mutant. **(E)** Survival curves of overall survival in p53 mutants with lymph-node-positive breast cancer.

Previous studies indicated that breast cancer cell line BT474 would undergo epithelial mesenchymal transition (EMT) and possess triple negative phenotype (BT474-PTEN-LTT, LTT) after trastuzumab treatment^2^. Real-time PCR indicated that CPA4 mRNA expression was elevated and p53 levels were reduced in LTT cells (Figure 1A). To confirm the association between CPA4 and p53, we analyzed the its levels in the UALCAN database (http://ualcan.path.uab.edu), including those of breast cancer subclasses. We used the Cancer Genome Atlas (TCGA; cBioPortal Breast Cancer: METABRIC, Nature 2012 and Nat Commun 2016: http://www.cbioportal.org) to analyze CPA4 levels in breast cancer patients. The results revealed that CPA4 was over-expressed in HER2-positive and TNBC patients (Figure 1B). Further statistical significance was observed in the basal-like 2 (BL2), mesenchymal (M) and unspecified (UNS) subgroups of TNBC (Figure 1C). TP53 mutation status showed that CPA4 mRNA levels were upregulated in the TP-mutant subgroup (Figure 1D). Next, we studied the correlation between CPA4 mRNA expression and prognosis using a Kaplan-Meier plot (www.kmplot.com). Kaplan-Meier survival analysis indicated decreased overall survival in patients whose tumors expressed higher CPA4 levels (Figure 1E). These data indicated that CPA4 was highly expressed in breast cancer tissues, especially those of TNBC with TP53 mutation.

### Expression of CPA4 in breast cancer tissues

**Figure 2:**
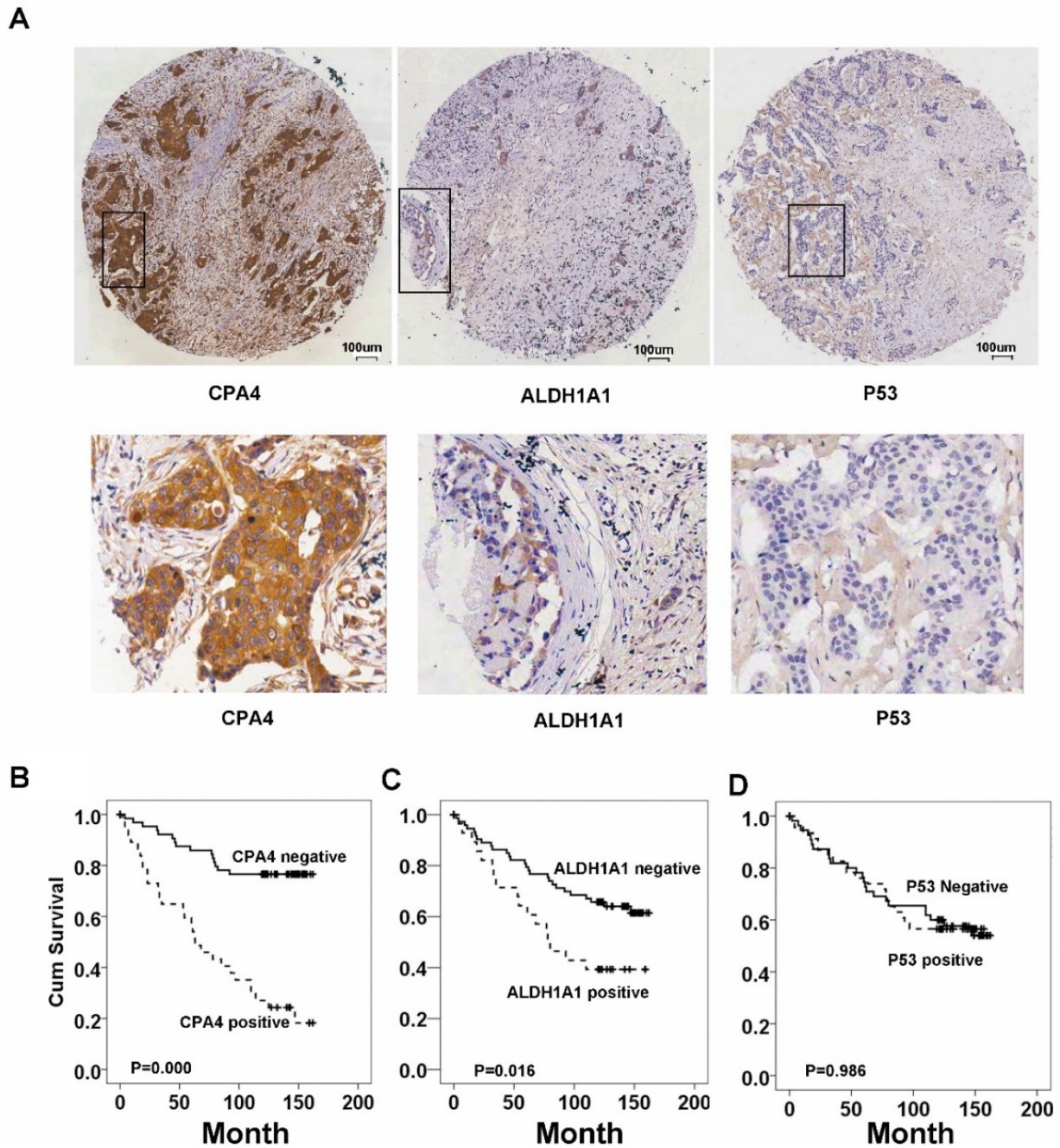
** CPA4, p53 and ALDH1A1 expressions in breast cancer tissues were determined by immunochemistry, Kaplan-Meier survival curve analysis, and the log-rank test. (A)** Positive expression of CPA4, ALDH1A1 and p53. **(B)** Survival curves for breast cancer patients. Overall survival curves for patients with negative CPA4 expression (solid line) and patients with positive CPA4 expression (dotted line). **(C)** Overall survival curves for patients with ALDH1A1 expression (solid line) and patients without ALDH1A1 expression (dotted line). **(D)** Overall survival curves for patients with negative p53 expression (solid line) and patients with positive p53 expression (dotted line).

Immunohistochemistrical analysis demonstrated that CPA4 was highly expressed in 32.1% of breast cancer samples (45/140), but weak or no staining of CPA4 occurred in the adjacent normal tissues (Figure 2A). Statistical analysis also indicated that positive staining for CPA4 was significantly associated with TNBC phenotype, lymph node metastasis and stage, while it was not significantly correlated with age, grade or depth of invasion (Table 1).

### Correlation between CPA4, p53 and ALDH1A1 in breast cancer tissues

To further validate the results of public datasets, we evaluated the expression of p53 and ALDH1A1 in the same tissue array by IHC. The results showed that the positive rate of ALDH1A1 was 25% (35/140), and positive staining of p53 was detected in 50% of breast cancer samples (70/140) (Table 1). Spearman correlation analysis also revealed that aberrant expression of CPA4 expression was positively associated with ALDH1A1 (P<0.01) and negatively correlated with p53 (P<0.05) in breast cancer tissues (Table 2). These observations demonstrated that over-expression of CPA4 might play important roles in cancer stemness.

### CPA4 and ALDH1A1 expression was associated with poor overall survival in breast cancer patients

**Figure 3:**
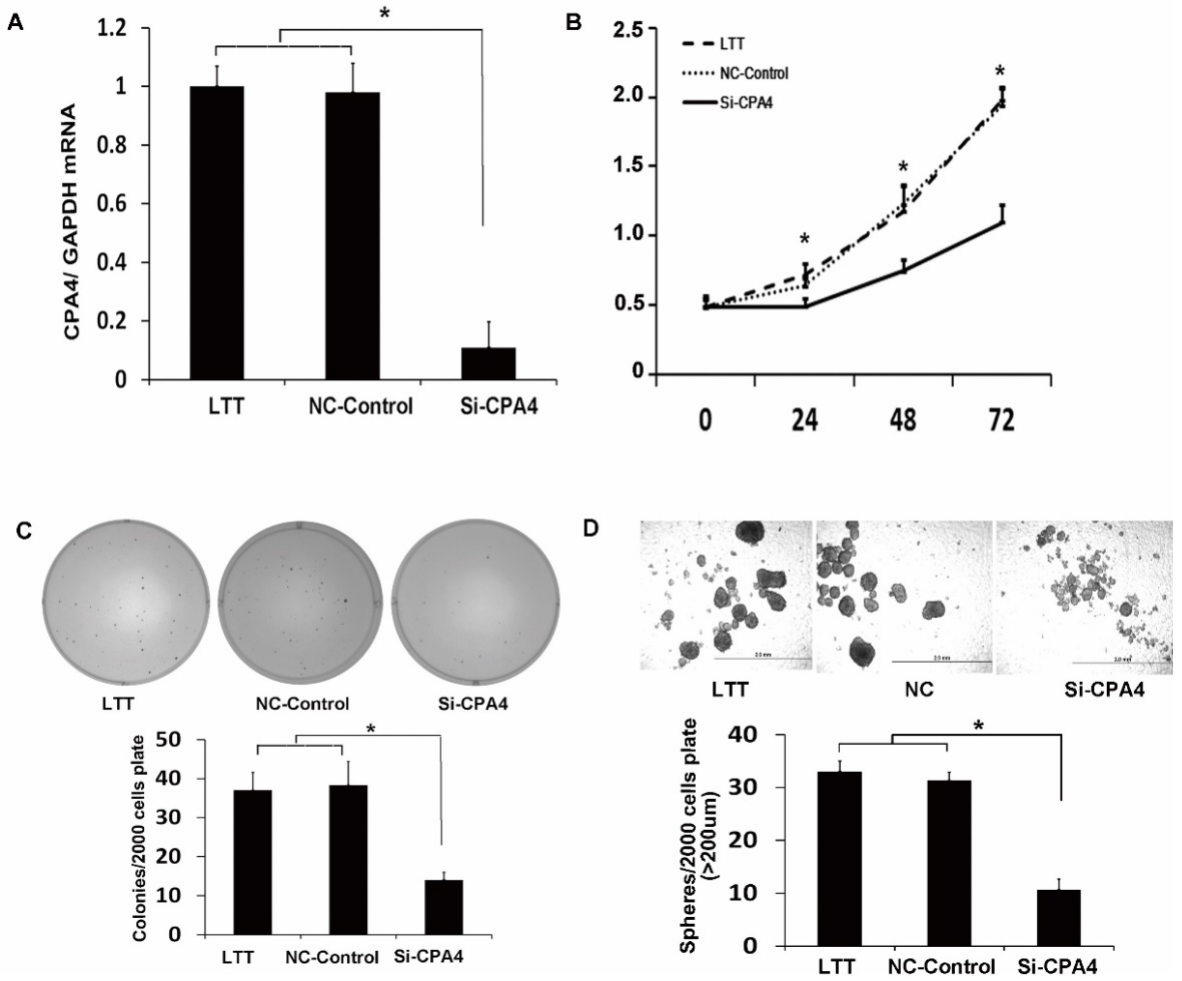
** CPA4 functionally regulated breast cancer cell proliferation and breast cancer stem cell characteristics *in vitro*. (A)** Real-time PCR analysis of CPA4 mRNA expression relative to GAPDH in transfection reagent (vehicle), non-targeted siRNA control (NT-control), or siRNA for CPA4 (siCPA4)-treated BT474 PTEN-LTT cells. **(B)** Cell proliferation following siRNA knockdown of CPA4 over 72 hours as determined via MTS proliferation. **(C)** Representative images of colonies formed 14 days after siRNA knockdown of CPA4 in LTT cells compared with the vehicle and NT-control treatments. **(D)** Representative images of mammospheres formed 14 days after siRNA knockdown of CPA4 in LTT cells compared with the vehicle and NT-control treatments.

Based on the association between CPA4 and ALDH1A1 or p53, we analyzed their effects on breast cancer patient survival. Kaplan-Meier survival analysis showed that breast cancer patients with high levels of ALDH1A1 or CPA4 were significantly correlated with poor overall survival (Fig.2). There was no significant correlation between high levels of p53 and poor survival (Fig.3).

### CPA4 silencing suppresses self-renewal ability and proliferation of breast cancer cell *in vitro*

To study the function of CPA4 in LTT cells, we used siRNA knockdown, which reduced the CPA4 expression by 90% (Figure 3A). We examined the effects of CPA4 knockdown on breast cancer proliferation via MTS assay. The results indicated that suppression of CPA4 in LTT cells significantly suppressed cancer cell growth (Figure 3B). We next studied the effect of CPA4 knockdown on *in vitro* tumorigenicity using colony-formation assays in soft agar. Colony-formation rates following CPA4 siRNA treatment were reduced to 62.2% compared with those of the controls in LTT cells (Figure 3C). The role of CPA4 in breast cancer cell self-renewal ability was determined using the mammosphere formation in serum-free suspension culture. CPA4 knockdown dramatically suppressed mammosphere formation by 75.8% in LTT cells, and the mammosphere size was also significantly decreased (Figure 3D). These results indicated that CPA4 may play functional roles in breast cancer stemness maintenance.

## Discussion

Breast cancer is the most commonly diagnosed cancer in women in China^11^. From its molecular and genetic characteristics, breast cancer is divided into several subtypes, among which, TNBC is the most aggressive and lacks effective treatment. In 2011, TNBC was divided into 7 subtypes: basal-like 1 (BL1), basal-like 2 (BL2), mesenchymal(M), mesenchymal stem-like (MSL), immunomodulatory (IM), luminal androgen receptor (LAR) and unstable (UNS) according to gene expression profiling^12^, ^13^. p53 were always deleted in TNBC tissues, and this deletion was associated with a poor prognosis^14^, ^15^.

CPA4, a metallocarboxypeptidase, catalyzes carboxy-terminal amino acids from the end of protein. Previous studies in our lab demonstrated that CPA4 was upregulated in BT474-PTEN-LTT cells but minimally detected in BT474 cells^3^. We next reported that CPA4 was significantly elevated in pancreatic cancer, gastric cancer, colorectal cancer and esophageal squamous cell carcinoma tissues^3^, ^4^, ^6^-^8^. Other studies confirmed that CPA4 was elevated in several cancer tissues and played functional roles in cancer progression^16^, ^17^. Handa et al. reported that CPA4 might be a promising therapeutic target in TNBC with aggressive phenotypes^18^. However, the correlation between CPA4 and p53 in TNBC tissues and its roles in the stemness phenotype remain unclear.

In this study, over-expression of CPA4 was observed in TNBC-distinct subgroups, including the BL2, M and UNS subgroups. Interestingly, CPA4 mRNA levels were also upregulated in the TP53-mutant subgroup. To further validate the CPA4, p53 and ALDH1A1 levels in breast cancer tissues, we examined their levels in breast cancer and adjacent normal tissues via immunohistochemistry. CPA4 levels were elevated in 32.1% of the breast tissue samples (45/140), and positive staining for CPA4 was statistically significantly associated with lymph node metastasis and stage. ALDH1 is one of the most widely used markers for identifying CSCs in many cancers^19^, ^20^. We next evaluated ALDH1A1 and p53 expression in the same tissue array via immunohistochemistry. Positive staining of ALDH1A1 occurred in 25% of the samples (35/140), and p53 staining occurred in 50% of the breast cancer tissues (70/140). Spearman's rank correlation revealed that aberrant expression of CPA4 was positively associated with ALDH1A1 (P<0.01) and negatively correlated with p53 (P<0.05) in breast cancer tissues. Kaplan-Meier survival analysis showed that CPA4 and ALDH1A1 over-expression was significantly correlated with overall survival of breast cancer patients. From the point of clinical application, high levels of CPA4 was found in TNBC tissues, and it could be measured as a potential serum biomarker for TNBC. Next, it was worthwhile to test the levels of CPA4 in breast cancer serum samples, and studied its diagnostic efficacy or prognosis value in TNBC.

Furthermore, CPA4 was demonstrated to be a functional gene and may promote breast cancer progression. We used siRNA to knockdown CPA4 levels in LTT cells to assess CPA4's effects on cell stemness *in vitro*. CPA4 downregulation significantly reduced lung cancer cell LTT proliferation, colony-formation assays in soft agar and mammosphere formation in serum-free medium. In future studies, we will further identify the molecular mechanisms of CPA4 in breast cancer progression. According to the above results, we speculated that CPA4 might help to establish the cancer microenvironment to facilitate cancer progression by catalyzing the substrate. It was possible that function-blocking agents targeting CPA4 might inhibit TNBC progression.

In conclusion, this is the first report to show that CPA4 was negatively correlates with p53 expression and inhibition of CPA4 could reduce the number of breast cancer cells with stemness property. It might be a potential therapeutic target for treating TNBC. However, further studies are needed to clarify the role of CPA4 in breast cancer with p53 deletion.

## Figures and Tables

**Table 1:** Correlation between CPA4 expression and clinicopathological characteristics in 140 breast cancer cases

	CPA4		
	negative	positive	p-value
**Age**	52.94±12.48	54.82±12.24	0.291
**Grade**			0.110
1+2	29	8	
3	66	37	
**Depth of invasion**			0.126
T1	24	6	
T2	64	32	
T3	7	7	
**Lymph node involvement**			**0.021**
N0	40	14	
N1	33	9	
N2	18	17	
N3	4	5	
**Stage**			**0.004**
Ⅰ	12	1	
Ⅱ	59	21	
Ⅲ	24	23	
Ⅳ	95	45	
**ER**			0.083
Negative	30	21	
Positive	65	24	
**PR**			0.321
Negative	38	22	
Positive	57	23	
**HER2**			0.393
Negative	68	29	
Positive	27	16	
**TNBC**			**0.021**
no	73	26	
yes	22	19	
**P53**			**0.019**
Negative	41	29	
Positive	54	16	
**ALDH1A1**			**0.000**
Negative	80	25	
Positive	15	20	

**Table 2:** Correlations between the Levels of CPA4, P53 and ALDH1A1

		P53	ALDH1A1
**CPA4**	**Spearman Correlation**	-0.199*	0.309**
	**Sig. (2-tailed)**	0.019	0
	**N**		

**. Correlation is significant at the 0.01 level (2-tailed). *. Correlation is significant at the 0.05 level (2-tailed).
